# Development of a Mobile App to Support Self-management of Anxiety and Depression in African American Women: Usability Study

**DOI:** 10.2196/24393

**Published:** 2021-08-17

**Authors:** Terika McCall, Muhammad Osama Ali, Fei Yu, Paul Fontelo, Saif Khairat

**Affiliations:** 1 Center for Medical Informatics Yale School of Medicine New Haven, CT United States; 2 Division of Health Informatics Department of Biostatistics Yale School of Public Health New Haven, CT United States; 3 Carolina Health Informatics Program University of North Carolina at Chapel Hill Chapel Hill, NC United States; 4 School of Information & Library Science University of North Carolina at Chapel Hill Chapel Hill, NC United States; 5 Health Sciences Library University of North Carolina at Chapel Hill Chapel Hill, NC United States; 6 National Library of Medicine Bethesda, MD United States; 7 School of Nursing University of North Carolina at Chapel Hill Chapel Hill, NC United States; 8 Cecil G. Sheps Center for Health Services Research University of North Carolina at Chapel Hill Chapel Hill, NC United States

**Keywords:** African Americans, women, mental health, anxiety, depression, telemedicine, mHealth, mobile applications, digital health, user-centered design, mobile phone

## Abstract

**Background:**

Anxiety and depressive disorders are the most common mental health conditions among African American women. Despite the need for mental health care, African American women significantly underuse mental health services. Previous mobile health studies revealed significant improvements in anxiety or depressive symptoms after intervention. The use of mobile apps offers the potential to eliminate or mitigate barriers for African American women who are seeking access to mental health services and resources.

**Objective:**

This study aims to evaluate the usability of the prototype of an app that is designed for supporting the self-management of anxiety and depression in African American women.

**Methods:**

Individual usability testing sessions were conducted with 15 participants in Chapel Hill, North Carolina. Cognitive walkthrough and think-aloud protocols were used to evaluate the user interface. Eye-tracking glasses were used to record participants’ visual focus and gaze path as they performed the tasks. The Questionnaire for User Interface Satisfaction was administered after each session to assess the participants’ acceptance of the app.

**Results:**

Participants rated the usability of the prototype positively and provided recommendations for improvement. The average of the mean scores for usability assessments (ie, overall reactions to the software, screen, terminology and app information, learning, and app capabilities) ranged from 7.2 to 8.8 on a scale of 0-9 (low to high rating) for user tasks. Most participants were able to complete each task with limited or no assistance. Design recommendations included improving the user interface by adding graphics and color, adding a tutorial for first-time users, curating a list of Black women therapists within the app, adding details about tracking anxiety and depression in the checkup graphs, informing users that they can use the talk-to-text feature for journal entries to reduce burden, relabeling the mental health information icon, monitoring for crisis support, and improving clickthrough sequencing.

**Conclusions:**

This study provides a better understanding of user experience with an app tailored to support the management of anxiety and depression for African American women, which is an underserved group. As African American women have high rates of smartphone ownership, there is a great opportunity to use mobile technology to provide access to needed mental health services and resources. Future work will include incorporating feedback from usability testing and focus group sessions to refine and develop the app further. The updated app will undergo iterative usability testing before launching the pilot study to evaluate the feasibility and acceptability of the prototype.

## Introduction

### Background

Approximately 1 in 4 African American women in the United States has experienced mental illness [1]. Furthermore, anxiety and depressive disorders are the most common mental health conditions among African American women [2]. However, African American women significantly underuse mental health services compared to their White counterparts (12.8% vs 28.7%, respectively) [1]. Historically, mental illness has been underreported in the African American community [3,4]; therefore, the true burden may be significantly higher than the reported prevalence estimates.

Data from the 2019 *National Survey on Drug Use and Health* [1] showed that approximately 30% of non-Hispanic Black women who reported experiencing mental illness in the past year did not receive mental health treatment during that time. Barriers such as the stigma of mental illness, limited access to treatment, lack of or inadequate health insurance, mistrust of providers, and limited health literacy (LHL) all prevent traditionally marginalized populations from seeking care [5-7]. A recent survey of 395 African American women [8] revealed that the most common reasons for not seeking mental health treatment or counseling when needed were attributed to cost, not knowing where to go to access services, lack of time, and stigma. The use of mobile apps may help to eliminate or mitigate barriers by providing information on affordable options for mental health care, facilitating connections with preferred therapists, eliminating travel time using remote services, and reducing potential stigma by providing a discreet way to receive care in a preferred setting (eg, in the privacy of their home).

Numerous interventions have successfully used apps to help participants in reducing their anxiety or depressive symptoms [9-12]. Two meta-analyses of randomized controlled trials exploring the use of smartphone mental health interventions to reduce anxiety or depressive symptoms [13,14] revealed that participants experienced a significant reduction in anxiety or depressive symptoms postintervention. However, the majority of published studies were conducted with a predominantly White sample, which may affect the generalizability of the results to other racial and ethnic groups. Furthermore, a study by Sarkar et al [15], which investigated the usability of commercially available apps for depression, found that, “while patients express interest in using technologies for self-management, current tools are not consistently usable for diverse patients.” The results of a recent systematic review [16] found only 3 studies focused on culturally informed telehealth interventions for managing anxiety and depression in African American adults [17-19] and only 2 included women [17,18]. Moreover, findings from focus group interviews revealed that African American women desired a culturally informed mental health app that can address their specific needs and preferences (eg, information to find a Black woman therapist) [8]. Previous studies have shown that African American women are comfortable with participating in mobile health (mHealth) research and interventions [20,21], and 80% of African American women own smartphones [22]. On average, they spend 19 hours per week on smartphone apps [22]. Therefore, there is a great opportunity to use apps to increase access to culturally informed resources and services for supporting the management of anxiety and depression in African American women, which is a significantly underserved population.

Recent years have witnessed a growing awareness of the effectiveness of using apps for psychological interventions. This interest includes studies on both the mental health benefits and usability of apps [23]; however, scientific evidence does not support the effectiveness of most mental health apps in the market [24]. Furthermore, many apps do not incorporate any evidence-based practices or clinical expertise regarding their usability [25]. An analysis of the user reviews for mental health apps taken from the App Store and Google Play revealed that the major issues with the apps were bugs and a poor user interface design [26]. Poor usability of mental health apps contributes to low engagement [26,27]. 

### Objective

The purpose of this study is to evaluate the usability of a prototype for an app designed for supporting the self-management of anxiety and depression in African American women. Specifically, participants evaluated the user interface on how well it helped them to complete basic tasks (eg, finding information about therapists). The findings will guide the further development of the app.

## Methods

### Prototype Development

The efficacious components of apps for anxiety and depression have been widely cited in the literature. Previous studies have highlighted the need for educational, psychotherapy, self-tracking, and personal development components in a mental health app that is designed to help users manage anxiety or depression [24,28-31]. Users who engaged in self-tracking and goal-setting experienced reduced depressive symptoms [30]. Furthermore, apps that offer guidance, such as personalized feedback and supportive messages, have been shown to have a positive effect on mental health outcomes [13,32].

**Figure 1:**
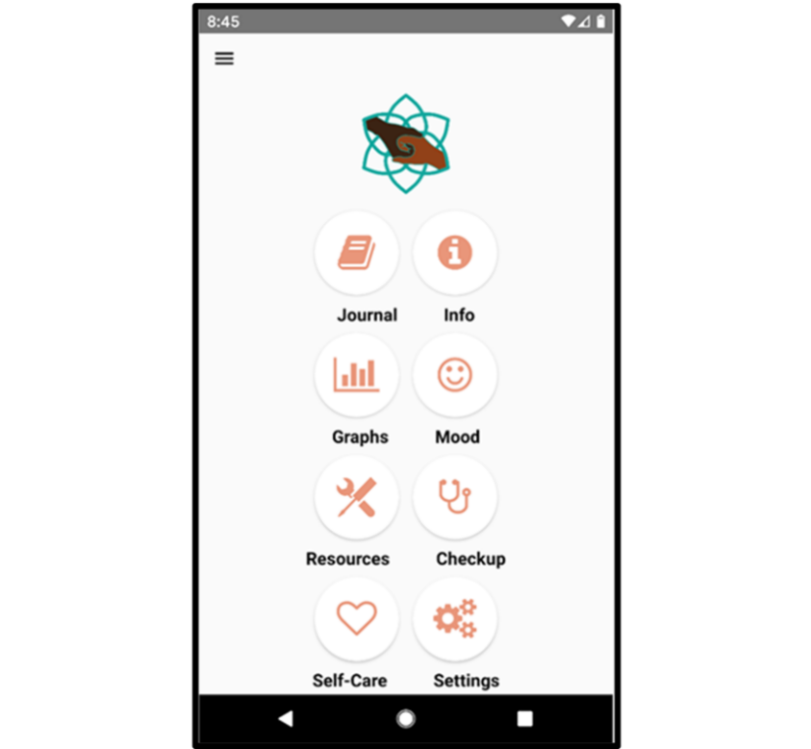
Screenshot of the app home screen.

The mental health app evaluated in this study was developed by a multidisciplinary team at the University of North Carolina (UNC) at Chapel Hill with expertise in counseling psychology, user experience and user interface design, mobile app development, and health informatics. The initial prototype, a native app for Android devices, included basic features informed by a review of the literature and a survey of mental health and wellness apps available in the App Store and Google Play. The primary features (Textbox 1) included a guided thought journal, information about anxiety and depression (including facts about the prevalence of anxiety and depression among African American women), self-assessments for depression (using questions from the Patient Health Questionnaire 9-item scale [33]) and anxiety (using questions from the Generalized Anxiety Disorder 7-item scale [34]), mood rating, graphs to track trends in depression and anxiety severity and mood rating history, culturally informed resources (eg, links to the Therapy for Black Girls therapist directory and podcast), and a self-care planner. Figure 1 shows a screenshot of the app’s home screen.

**Description of primary app features.**

**Journal**

- A guided thought journal that allows the user to record their thoughts and feelings, and if applicable, prompts them to think about future actions and displays a supportive message.

**Info**

- Provides information about anxiety and depression (including facts about the prevalence of anxiety and depression among African American women). The user is also presented with information about symptoms, causes, treatments, and tips for managing anxiety or overcoming depression.

**Checkup**

- Allows the user to complete self-assessments to screen for the presence and severity of depression using questions from the Patient Health Questionnaire 9-item scale, and anxiety using questions from the Generalized Anxiety Disorder 7-item scale.

**Mood**

- Displays a Likert scale of images (emoticons) for users to rate how they feel.

**Graphs**

- Allows the user to track trends in their anxiety and depression severity, and mood rating history.

**Resources**

- Presents a library of culturally informed resources that links users to mental health information, therapists’ directories (eg, Therapy for Black Girls), mental health and wellness podcasts (eg, Balanced Black Girl), financial assistance, and suicide crisis information.

**Self-care planner**

- Allows the user to create a self-care plan and checks in with the user after the chosen end date to see if the activities were completed.

### Recruitment

The usability study was exempted from a full review by the UNC at Chapel Hill’s institutional review board. Prior research showed that 5 participants could reveal about 85% of the problems in a formative usability study [35,36]. In addition, findings from a notable study by Virzi [37] showed that 80% of usability problems were detected in 4 or 5 participants. A total of 15 participants were recruited to test the usability of the app. Participants were recruited via posts on social media (eg, Facebook and Twitter), a recruitment listing on Research for Me @UNC, and flyers posted in the Durham and Chapel Hill communities inviting women (18 years or older) who identified as Black or African American or multiracial (ie, Black or African American and another race) and had a history of anxiety or depression to participate in the study. However, study participation did not require a clinical diagnosis of an anxiety or depressive disorder. Each participant received a US $25 gift card for their completion of the study.

### Procedures

In February and March 2020, individual usability testing sessions were held for each participant at the UNC at Chapel Hill School of Nursing’s Biobehavioral Lab. Before the start of usability testing, a researcher went through consent forms with participants and obtained their signatures. In addition, participants were informed that the study would last approximately 1 hour.

First, the participants received a brief overview of the study aims and a description of the Tobii eye-tracking glasses and software (Tobii Pro AB) [38]. The Tobii Pro 2 glasses were calibrated for each participant before beginning the usability testing. Participants were provided with an Android mobile phone (ie, Google Pixel 2), and were assigned a persona (ie, a fictional character created to represent a target user) and scenario (ie, a fictitious story about a target user and their motivation for using the app; Multimedia Appendix 1). Then, they were asked to perform a series of 4 tasks in the app and think aloud as they completed them. To reduce participants’ workload while testing most of the app’s features, the study sample was split and participants were assigned either scenario 1 or scenario 2 (Textbox 2). The instructions for each task included fictitious information for the participants to enter so that they could complete the task. No personal information was solicited or involved during the interaction between the participants and the app.

**List of tasks for scenarios (differences between the scenarios have been italicized).**

**Scenario 1 tasks**

- Find out your levels of *anxiety* for the past 6 weeks.
- Find information on how to *manage anxiety.*
- *Add a new entry to your journal.*
- Locate a therapist to schedule an appointment.

**Scenario 2 tasks**

- Find out your levels of *depression* for the past 6 weeks.
- Find information on how to *overcome depression.*
- Create a *self-care* plan.
- Locate a therapist to schedule an appointment.

The cognitive walkthrough method [39] was used to evaluate the user interface design on how well it supported users in learning to complete tasks. Specifically, this method was used to “evaluate the ease with which users can perform a task with little or no formal instruction or informal coaching” [39]. Participants were told to speak aloud their thoughts and actions so that they could be recorded using the Tobii software. The researcher read a persona, scenario, and the first task to the participant and then instructed them to begin. This process was repeated for tasks 2-4. The Tobii software recorded the videos of participants’ interactions with the app while they completed the tasks, including taps on the phone screen, eye movements, and the amount of time spent on each task.

### Measures

The usability of the app was measured by participants’ ability to complete tasks efficiently (measured by time and number of taps to complete each task) and their satisfaction with the user interface (measured by scores in each domain of the Questionnaire for User Interface Satisfaction [QUIS]; [40]).

#### Benchmarks for Cognitive Walkthrough Tasks

Benchmarks comprising a list of actions that should be performed to complete each task efficiently were created by the research team. Each task was divided into steps that should be taken to complete the actions along the *happy path* (ie, the most efficient sequence of steps to produce the desired outcome). The benchmarks were used to evaluate the participants’ actions while completing the tasks (Multimedia Appendix 2).

#### The Questionnaire for User Interface Satisfaction

After the usability testing was completed, each participant was given a hardcopy of an adapted version of the QUIS [40] for further assessment. In the QUIS, the words *system* and *computer* were replaced with *app* in sections of the instrument. The QUIS “measures the user’s subjective rating of the human-computer interface” [40]. The five domains of the QUIS covered (1) overall reaction to the software (eg, How easy was the app to use? How stimulating was the app?), (2) screen (eg, How easy was it to read the characters on the screen? How clear was the organization of information on the screen?), (3) terminology and app information (eg, How consistent was the use of terms throughout the app? How clear were the messages on the screen that prompted input from the user?), (4) learning (eg, How easy was it to explore new features through trial and error? How often could tasks be performed in a straightforward manner?), and (5) app capabilities (eg, How fast was the app? How easy was it to correct your mistakes?). Response options for each question were displayed on a Likert scale ranging from low to high (scores of 0-9). All 27 questions were weighted equally and collapsed into 5 mean scores, one for each domain and for each individual participant. The minimum and maximum mean scores, average of means, and SDs were calculated for each domain. Participants were informed to select *N/A* (not applicable) for the survey items that were not applicable. The QUIS also included 2 qualitative questions that asked participants to list the most positive and negative aspects of the app.

### Statistical Analysis

#### Quantitative Data Analysis

Descriptive statistics were calculated as means, SDs, and ranges for continuous variables (eg, age) and as frequencies and percentages for categorical variables (eg, education level) for sample characteristics. To measure efficiency, the mean, SD, and range were calculated for the time required to complete each task and the number of taps needed to complete each task. To measure user interface satisfaction, the means, SDs, and ranges for scores in each QUIS domain were calculated. Statistical analyses were conducted using SPSS (version 26, IBM Corp) [41].

#### Qualitative Data Analysis

The results of the cognitive walkthrough sessions were summarized qualitatively. The Tobii eye-tracking software produced heat maps that revealed the focus of the participants’ visual attention on the screens. Furthermore, the most positive and negative aspects of the app reported on the QUIS were summarized. Specifically, common issues with the app prototype that were identified were discussed, and positive aspects were highlighted.

## Results

### Participants

A total of 15 participants tested the usability of the app using the cognitive walkthrough and think-aloud methods. Participants were in the age range of 20-66 years (mean age 29.8 years, SD 12.4 years), and all identified as either Black or African American or multiracial (ie, Black or African American and another race) and female. Most participants (13/15, 87%) obtained a bachelor’s degree or higher and indicated that they used mobile apps 4 or more times per day (14/15, 93%). Table 1 summarizes the characteristics of the study participants.

**Table 1:** Characteristics of study participants (N=15).

Variables			Values
Age (years), mean (SD)			29.8 (12.4)
**Education, n (%)**			
	Less than a bachelor’s degree	2 (13)	
	Bachelor’s degree or higher	13 (87)	
**Mobile app use, n (%)**			
	1-3 times per day	1 (7)	
	4 or more times per day	14 (93)	

### Cognitive Walkthrough

#### Scenario 1 Tasks

After the persona and scenario were read to the participants (n=8), they were instructed to think aloud as they completed each of the 4 tasks for scenario 1. Table 2 provides a summary of the results for the cognitive walkthrough for scenario 1 tasks. Tasks included finding the recorded levels of anxiety for the past 6 weeks, finding information on how to manage anxiety, adding a new entry in the guided thought journal, and locating a therapist to schedule an appointment.

**Table 2:** Summary of results for the cognitive walkthrough for scenario 1 tasks (n=8).

Task	Completed, n (%)	Benchmark			Participant outcomes	
		Time	Taps, n	Time, range		Taps, range
Find out your levels of anxiety for the past 6 weeks	8 (100)	13 seconds	3	20 seconds to 1 minute 41 seconds		3-6
Find information on how to manage anxiety	8 (100)	25 seconds	6	48 seconds to 4 minutes 24 seconds		6-37
Add a new entry to your journal	5 (63)	3 minutes 10 seconds	15	2 minutes 48 seconds to 6 minutes 10 seconds		17-19
Locate a therapist to schedule an appointment	8 (100)	56 seconds	8	1 minute 42 seconds to 3 minutes 21 seconds		10-35

Most participants were able to complete each task fully with limited or no assistance. On average, participants took longer to complete the tasks than the benchmark times. Adding a new entry to the journal proved to be the most cumbersome task as it required participants to be thoughtful when they entered text and selected their feelings from a list. Two participants had difficulty with locating the information on how to manage anxiety, searching in the resources section of the app instead of information. To locate a therapist, participants were required to tap a button that was linked to a website with a directory of primarily Black women therapists. Although all participants were able to complete the task of locating a therapist, many reported that the interface of the website was not mobile friendly and required a lot of scrolling to find out if the therapist was accepting new clients. The actions completed, number of taps, and the amount of time spent on each task were recorded for each participant in Multimedia Appendix 3.

#### Scenario 2 Tasks

Following the protocol, the persona and scenario were read to the participants (n=7). Next, they were instructed to think aloud as they completed each of the 4 tasks. Table 3 provides a summary of the results for the cognitive walkthrough for scenario 2 tasks. Tasks included finding the recorded levels of depression for the past 6 weeks, finding information on how to overcome depression, creating a plan for self-care, and locating a therapist to schedule an appointment.

**Table 3:** Summary of results for the cognitive walkthrough for scenario 2 tasks (n=7).

Task	Completed, n (%)	Benchmark			Participant outcomes	
		Time	Taps, n	Time, range		Taps, range
Find out your levels of depression for the past 6 weeks	7 (100)	14 seconds	3	27 seconds to 48 seconds		3-6
Find information on how to overcome depression	7 (100)	20 seconds	6	28 seconds to 2 minutes 14 seconds		6-14
Create a self-care plan	7 (100)	1 minute 17 seconds	15	1 minute 17 seconds to 2 minutes 47 seconds		16-23
Locate a therapist to schedule an appointment	7 (100)	49 seconds	8	1 minute 7 seconds to 2 minutes 9 seconds		9-13

All participants were able to determine their depression levels for the past 6 weeks and reported the most recent level of depression recorded in the graph. One participant had difficulty with locating the information on how to overcome depression and searched in the resources section of the app instead of the information feature. The participants liked the ability to track their self-care. The time to complete a self-care plan varied according to the typing speed of the participants. The actions completed, number of taps, and the amount of time spent on each task were recorded for each participant in Multimedia Appendix 4.

### QUIS Scores

#### Scenario 1 Tasks

**Figure 2:**
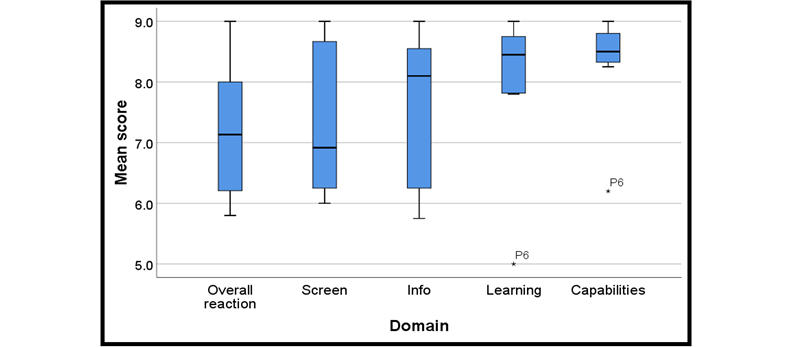
Boxplots for the 5 domains of the Questionnaire for User Interface Satisfaction for scenario 1 tasks. P6: participant #6.

The average of mean scores for each domain ranged from 7.2 to 8.3 on a scale of 0-9 (low to high rating; Table 4). The average of mean scores for the overall reaction to the software was 7.2 (SD 1.1), that for screen was 7.3 (SD 1.3), that for terminology and app information was 7.6 (SD 1.3), that for learning was 8.0 (SD 1.3), and that for app capabilities was 8.3 (SD 0.9). Figure 2 displays a boxplot of the mean scores for the 5 domains of the QUIS for scenario 1 tasks. There was 1 outlier (participant #6) with a score of 5.0 for the learning domain and 6.2 for app capabilities.

**Table 4:** Summary of mean scores for the 5 domains of the Questionnaire for User Interface Satisfaction for scenario 1 tasks (n=8).

Domain	Mean scores, range	Average of mean scores, mean (SD)
Overall reaction to the software	5.8-9.0	7.2 (1.1)
Screen	6.0-9.0	7.3 (1.3)
Terminology and app information	5.8-9.0	7.6 (1.3)
Learning	5.0-9.0	8.0 (1.3)
App capabilities	6.2-9.0	8.3 (0.9)

#### Scenario 2 Tasks

**Figure 3:**
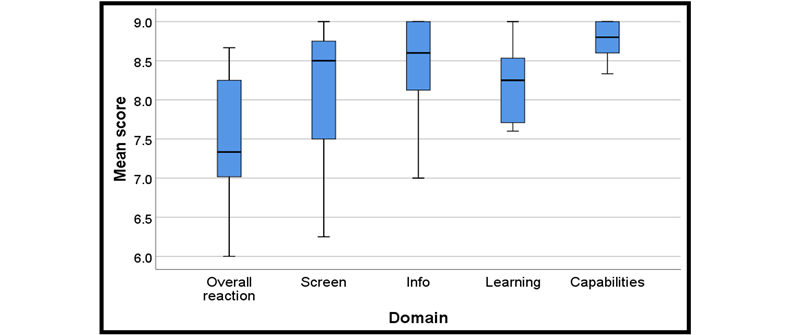
Boxplots for the 5 domains of the Questionnaire for User Interface Satisfaction for scenario 2 tasks.

The average of mean scores for each domain ranged from 7.5 to 8.8 on a scale of 0-9 (low to high rating; Table 5). The average of mean scores for the overall reaction to the software was 7.5 (SD 1.0), that for screen was 8.0 (SD 1.0), that for terminology and app information was 8.4 (SD 0.8), that for learning was 8.2 (SD 0.5), and that for app capabilities was 8.8 (SD 0.3). Figure 3 displays a boxplot of the mean scores for the 5 domains of the QUIS for scenario 2 tasks.

**Table 5:** Summary of mean scores for the 5 domains of the Questionnaire for User Interface Satisfaction for scenario 2 tasks (n=7).

Domain	Mean score, range	Average of mean scores, mean (SD)
Overall reaction to the software	6.0-8.7	7.5 (1.0)
Screen	6.3-9.0	8.0 (1.0)
Terminology and app information	7.0-9.0	8.4 (0.8)
Learning	7.6-9.0	8.2 (0.5)
App capabilities	8.3-9.0	8.8 (0.3)

### Qualitative Results

Table 6 shows the participants’ feedback on the app design. Overall, participants felt that the app was easy to use, organized well, and had fast processing speed. General recommendations for improvement included adding more graphics and color, including instructions for data entry textboxes, a tutorial for first-time users, and crisis support monitoring. Regarding content, participants thought the information provided was of high quality, they also liked that links were provided to helpful outside resources (including a link to Black women therapists with their availability), and that graphs and text provided information to track their anxiety and depression severity. Participants recommended that a curated list of Black women therapists should be provided within the app to avoid linking externally to websites that are not mobile friendly (eg, required too much scrolling).

**Table 6:** Feedback on app design.

Themes	Positive feedback	Recommendations
Overall reactions to the app	Easy to use Organized well Fast processing speed Every screen had a header App saves information entered and indicates the completion of task Nice logo	Improve user interface by adding more graphics and color Add instructions under start and end date fields to explain what to enter in the textboxes Add a tutorial for first-time use Monitor for crisis support
Content	Quality of information Linked to helpful outside resources List of Black women therapists with their availability Graphs and text provide information to track anxiety and depression Tips to help manage anxiety	Curate a list of Black women therapists in the app to avoid linking externally to websites that are not mobile friendly
Features	Tracking anxiety and depression Self-care planning Guided thought journal	Add footnotes to anxiety and depression history graphs that it can be used to track trends Informing users that they can use the talk-to-text feature for journal entries to reduce burden
Navigation and error prevention	Icons were helpful for guidance on navigating the app Drop-down menu clearly lists all features Great prompts in the guided thought journal System uses highlighting to indicate the selection made System alerts when there is a mistake or missing information	Relabel information icon to make clear it contains mental health information Improve clickthrough sequencing

Anxiety and depression checkups, self-care planner, and guided thought journal features were rated positively. Recommendations for improvement included adding a footnote to anxiety and depression history graphs to indicate that it can be used to track trends and adding instructions to the journal feature for informing users that they can use the talk-to-text feature for journal entries to reduce burden. Regarding navigation and error prevention, participants thought that the icons were helpful for guidance on navigating the app and liked that the drop-down menu clearly lists all features. They also noted the great prompts in the guided thought journal. Participants recommended that the information feature be relabeled to elucidate that it contains mental health information and that clickthrough sequencing be improved.

### Heat Maps

**Figure 4:**
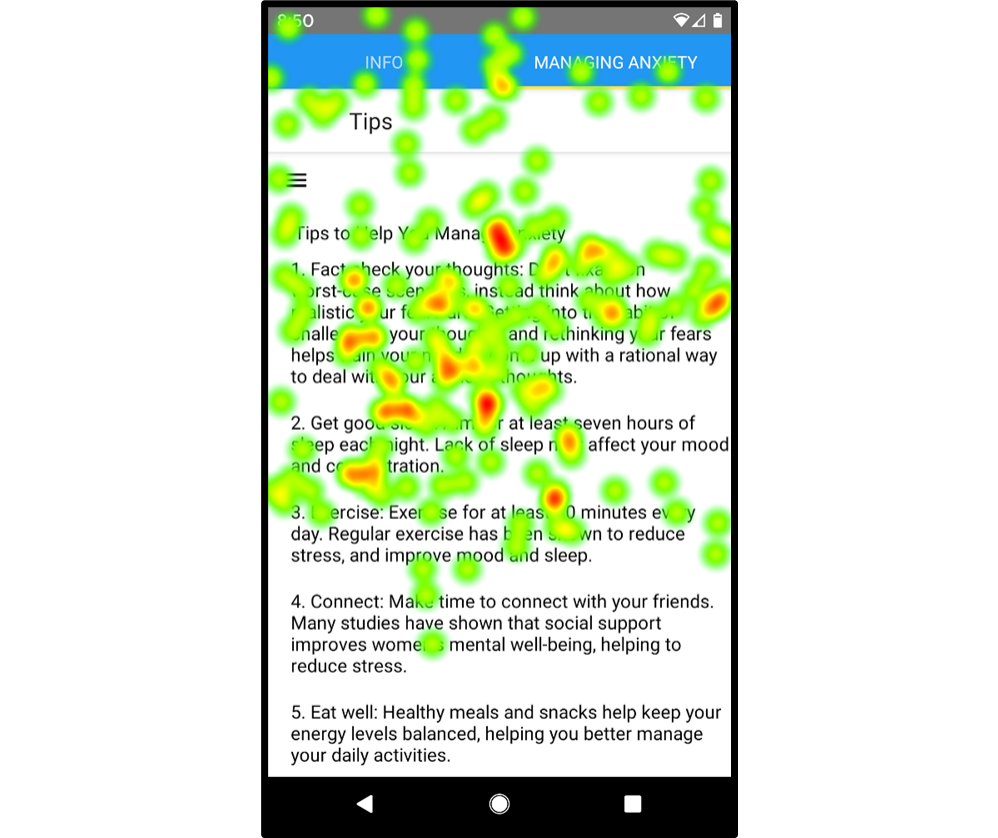
Heat map of the managing anxiety tips screen showing areas that participants focused on most.

**Figure 5:**
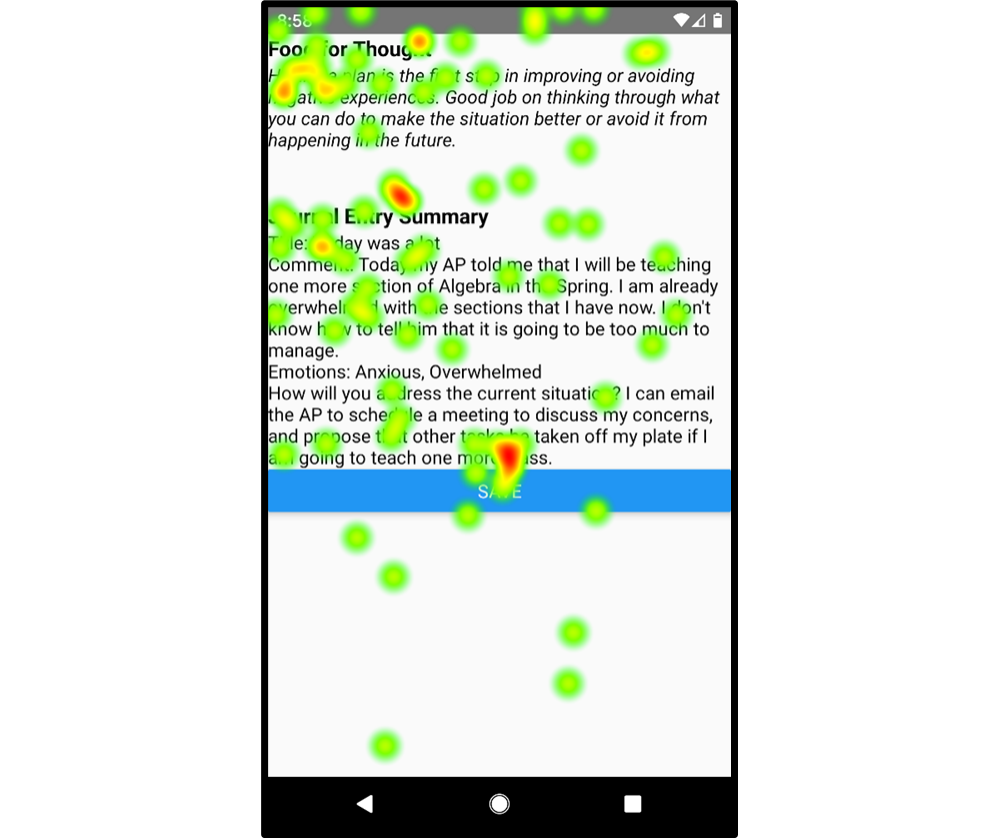
Heat map of the journal entry summary screen showing areas that participants focused on most.

The eye-tracking software showed that participants primarily focused on the left and middle areas of the screen while looking for information. The heat map for the managing anxiety tips screen (Figure 4) revealed that participants spent the most time viewing the title, left side, and middle of the screen (the areas of highest intensity). Similarly, Figure 5 shows that on the heat map of the journal entry summary screen, the titles, left side of the screen, and *Save* button were the areas where participants focused on most (the areas of highest intensity).

## Discussion

### Principal Findings

To our knowledge, this is one of the first mobile apps specifically designed to support the self-management of anxiety and depression in African American women, irrespective of physical health conditions or special circumstances. This is an important distinction because there are studies that focus on culturally informed mental health interventions for Black women; however, the participants had a particular physical health condition (eg, HIV positive) [18] or special circumstances (eg, caregivers of patients with dementia) [17]. Our results demonstrate that the participants were mostly satisfied with the user interface of the app prototype. Moreover, the average of means scores for the overall reaction to the software, screen, terminology, app information, learning, and app capabilities were high. They ranged from 7.2 to 8.3 on a scale of 0-9 (low to high rating) for scenario 1 tasks (Table 4), and 7.5 to 8.8 for scenario 2 tasks (Table 5). Most participants were able to complete each task fully with limited or no assistance. However, the variance in time to complete each task was primarily attributed to the amount of time it took for participants to talk through their thoughts and actions, differences in typing speed, or using an alternative method to search (eg, using a link from the hamburger menu instead of tapping the feature icon).

Furthermore, a few participants were unsure if they needed to first record their anxiety levels in the app before locating it in the graph and confused about where to find information about anxiety and depression, looking at the resources or self-care features of the app instead of the information feature. Participants expressed that they thought the information feature was the place to find information about the app and not about anxiety. Moreover, although the majority of the participants had no problem finding the button within the app that was linked to the website with the therapist directory, some participants expressed that the website itself was not mobile friendly and required a lot of scrolling to find out if the therapist was accepting new clients.

The aforementioned usability testing results produced the following considerations for the development of a mental health app to support the self-management of anxiety and depression in African American women: (1) the app should be intuitive and easy to use, (2) the app should include a feature to self-monitor mental health (eg, depression severity monitoring), (3) the app should allow users to learn coping skills (eg, tips on how to overcome depression), (4) the app should connect users with needed resources (eg, therapist), and (5) the app should provide users with the option to plan activities for self-care. In addition, the interface should be visually appealing and have clear labeling. These recommendations are consistent with those found in the literature that highlight the need for educational, psychotherapy, and personal development components in a mental health app designed to help users manage anxiety and depression [28,29]. Having a tutorial on how to use the app features and find content would also help improve user satisfaction and increase engagement. Furthermore, culturally tailoring the content and resources helps personalize the app to meet the specific needs and preferences of African American women.

The eye-tracking software demonstrated that the participants primarily focused on the left side and middle of the screen when looking for information. This is consistent with a previous study that showed that people spend 80% of their time looking at the left side of the screen [42]. Developers should consider placing important information on the left side or middle of the screen to make it easier for users to find. As external sites that the app links to may not be mobile friendly or have good usability, consider either only linking to mobile-friendly sites or placing all important content within the app. In addition, navigation buttons should be large and spaced well so that users with longer fingernails do not have difficulty tapping them. According to Fitts Law, the longer the distance to the target and the smaller the size of the target, the longer it takes to complete the movement [43]. Feedback from the usability testing sessions informed the current design and development of the app, in terms of both anesthetics (eg, layout, color scheme) and information architecture (eg, renaming the information feature).

Downloading an app does not necessarily mean that the user will continue to use it consistently in the long term. Past research conducted on the usage patterns of mental health apps showed that despite the high amount of initial installations and daily active minutes spent, only a small percentage of users continued to use the apps for longer than a couple of weeks [44]. There are several challenges in using apps for mental health care. Factors that affect the uptake include both the health and digital literacy of users. Approximately one-third of adults in the United States have LHL [45]. African American adults have a higher prevalence of LHL than their White counterparts [45]. Individuals with LHL are less likely to use digital devices for health-related purposes, which can hinder the use of smartphone mental health app interventions [46]. To promote adoption among users with LHL, smartphone health interventions should incorporate features that target a person’s own health literacy needs and technical skills [47].

Furthermore, poor usability in which an app may appear to be filled with bugs if a function or more does not seem to work, also affects the long-term use of the app. Another challenge is the lack of user engagement within apps that can cause app use to decline [27]. A previous case study on building a highly rated mental health app showed that users tend to prefer apps that focus on self-development and change rather than interventions from external sources [28]. The app tested in this usability study will promote engagement by prompting users to enter their mood daily. Users are also encouraged to record their thoughts and feelings in the guided thought journal for reflection and planning future actions. Push notifications will also remind users to complete the anxiety (Generalized Anxiety Disorder 7-item scale) and depression (Patient Health Questionnaire 9-item scale) checkups every 2 weeks to track progress. In addition, the app checks in with the user after the chosen end date to confirm whether activities in their self-care plan were completed. Tailored feedback is incorporated into some components of the app, such as the guided thought journal, mood tracking, and self-care plan.

When designing the user interface of the app itself, several variables should be considered. The experience of the user with app usage, their background, how they might function under stress while using the app, and the environment in which the app is being used are just some of the important factors that should be considered to ensure a positive user experience. A good mental health app should consider users’ need for support, sociocultural factors, and personal development goals. For example, an app focusing on providing anxiety management should educate its users about anxiety, provide culturally informed self-help options to manage their anxiety through low-intensity techniques, and give users the ability to track their progress and connect with preferred providers for additional support if necessary. Self-care apps should assume that their users are independent and provide support to them when needed [27]. A *one-size-fits-all* approach to designing mHealth interventions may lead to more options but continued disparity in receiving mental health care. The inequitable design of digital health tools further perpetuates the exclusion of underserved populations from mental health care [48]. Incorporating recommendations from intended users and knowledge of their technology use and behaviors can help mitigate potential intervention-generated inequalities [49].

One caveat is that the use of apps to receive mental health support may not be appropriate for everyone. If used as an adjunct to therapy, clients should be screened to determine whether the use of this modality is appropriate for treatment [50]. A survey of African American women revealed that video calls were an acceptable modality to communicate with a professional to receive help in managing anxiety or depression [20], whereas text messaging was not [51]. Therefore, an option to communicate with a mental health professional via video calls within the app may be a useful feature to include. The extent to which a mental health app is used may vary. It can be used for self-management only, peer support, the primary modality to receive mental health care from a professional (eg, telecounseling), or as an adjunct to in-person or other methods for remote counseling.

### Strengths and Limitations

The main strengths of this usability study are its rigorous design and use of the cognitive walkthrough and think-aloud method, eye-tracking technology-assisted usability evaluation, and administration of the QUIS to capture user performance, physiological data, and qualitative feedback completely. In addition, 15 participants were recruited to participate in the usability testing of the app, thus providing a more than adequate sample size.

One of the main limitations was that our participants were mostly younger women (under 50 years old) with at least a bachelor’s degree. This may limit the generalizability of the findings to older African American women and those with less than a bachelor’s degree. However, previously reported statistics revealed that younger Black women had a higher prevalence of lifetime anxiety (eg, generalized anxiety disorder) and mood disorders (eg, major depressive disorder) than older Black women (50 years or older) [2]. Although access to mental health services and resources may be less of an issue for African American women with at least a bachelor’s degree, access to culturally informed resources and a professional that meets the ethnicity and gender preferences of the patient may remain an issue as less than 5% of active psychologists in the United States are African American women [52].

In addition, the geographical restriction in recruiting participants may have resulted in the opinions and perceptions of the participants not reflecting those of a nationally representative sample. Another limitation was that this study did not focus on the efficacy of the app to reduce anxiety and depressive symptoms, as this would require a randomized controlled trial and significant resources to be done properly. Despite these limitations, the study yielded useful information that provides guidance for designing an app to help African American women and other populations in managing anxiety and depression.

### Conclusions and Future Directions

African American women have high rates of smartphone ownership (80%) [22], and there is a great opportunity to use mobile technology to provide mental health resources and services to them. Poor usability severely affects the engagement and effectiveness of mHealth interventions. Therefore, usability testing should be incorporated in the design and development of mental health apps to increase adoption, engagement, and user satisfaction. This study contributes to an improved understanding of users’ experiences with an app tailored to support the self-management of anxiety and depression in African American women, which is an underserved population. It is recommended that future researchers and app designers consider the proposed content, features, and considerations highlighted by this study while developing a mental health app for this population to enhance the user experience. We are planning to further develop the app by incorporating feedback from this study, and the new version will undergo iterative usability testing before the launch of the pilot study to evaluate the feasibility and acceptability of the prototype.

## Appendix

Multimedia Appendix 1 Usability testing scenarios and tasks.

Multimedia Appendix 2 Cognitive walkthrough benchmarks.

Multimedia Appendix 3 Usability testing participant performance versus benchmark for scenario 1 tasks.

Multimedia Appendix 4 Usability testing participant performance versus benchmark for scenario 2 tasks.

## Abbreviations

LHL: limited health literacy
mHealth: mobile health
QUIS: Questionnaire for User Interface Satisfaction
UNC: University of North Carolina
