# Supplementary material for: Development of a Mobile App to Support Self-management of Anxiety and Depression in African American Women: Usability Study
Source: JMIR Form Res. 2021 Aug 17;5(8):e24393. doi: 10.2196/24393 (PMC8408754; doi:10.2196/24393)
Supplement: Multimedia Appendix 1 [file formative_v5i8e24393_app1.pdf]

USABILITY TESTING SCENARIOS AND TASKS

Scenario 1 and Tasks

You are Betty Jackson a teacher at Miami Lakes Middle School in South Florida. You have been stressed out for the past 2 months due to demands at work and home. In the past, your doctor has recommended that you speak to a therapist, however you have been reluctant to schedule an appointment with the therapist you were referred to. You recently saw an advertisement for a new mental health app on Facebook and decided to download it on your mobile phone. You have been using the app for 6 weeks now, and you would like to review your anxiety severity over the last 6 weeks. You review the anxiety checkup graph to see your progress and discover that your anxiety has become progressively worse. This leads you to use the app to find information about managing anxiety, and locate a therapist in your area to schedule an appointment.

Task 1: Find out your levels of anxiety for the past 6 weeks. Let me know when you're done.

1. Open the app
2. Find the graphs feature
3. Find the anxiety checkup graph
4. Report your most recent level of anxiety recorded in the graph (severity and date)

Task 2: Find information on how to manage anxiety. Let me know when you're done.

1. Find the information feature
2. Find the anxiety information
3. Find the information on how to manage anxiety
4. Report the first tip on managing anxiety

Task 3: Add a new entry to your Journal. Let me know when you're done.

1. Find the journal feature
2. Create a new journal entry.
    a. **Title.** Enter the following title for the journal entry: "Today was a lot"
    b. **Describe your thoughts and how you felt.** Enter manually or use the voice-to-text feature to enter the following information:

        "Today my AP told me that I will be teaching one more section of Algebra in the Spring. I am already overwhelmed with the sections that I have now. I don't know how to tell him that it is going to be too much to manage."

    c. Choose "anxious" and "overwhelmed" as your options to report your feelings
    d. **Is there anything you can do to make the situation better, or avoid it from happening in the future?** Select "Yes"
    e. **What do you think can be done to make the situation better?** Enter manually or use the voice-to-text feature to enter the following information:

> “I can email the AP to schedule a meeting to discuss my concerns, and propose that other tasks be taken off my plate if I am going to teach one more class.”

Task 4: Locate a therapist to schedule an appointment. Let me know when you’re done.

1. Find the resources feature
2. Find a therapist in your area from the Therapy for Black Girls Therapist Directory
    a. Search by zip code “33015”
    b. Find “Nakita Charles” and report whether she is accepting new clients

Scenario 2 and Tasks

You are Candace Smith a student at Big State University in North Carolina. You have been feeling depressed for the past 2 months due to demands at school. In the past, your doctor has recommended that you speak to a therapist, however you have been reluctant to schedule an appointment with the therapist you were referred to. You recently saw an advertisement for a new mental health app on Twitter and decided to download it on your mobile phone. You have been using the app for 6 weeks now, and you would like to review your depression severity over the last 6 weeks. You review the depression checkup graph to see your progress and discover that your depression has become progressively worse. This leads you to use the app to find information about managing depression, and locate a therapist in your area to schedule an appointment.

Task 1: Find out your levels of depression for the past 6 weeks. Let me know when you're done.

1. Open the app
2. Find the graphs feature
3. Find the depression checkup graph
4. Report your most recent level of depression recorded in the graph (severity and date)

Task 2: Find information on how to overcome depression. Let me know when you're done.

1. Find the information feature
2. Find the depression information
3. Find the information on how to overcome depression
4. Report the first tip on overcoming depression

Task 3: Create a self-care plan. Let me know when you're done.

1. Find the self-care plan feature
2. Create a new self-care plan for the week of <u>March 23, 2020</u> to <u>March 29, 2020</u>.
    a. Enter the following activities into the plan.
        i. "Go to yoga class"
        ii. "Have lunch with Ashley"
        iii. "Read one chapter of the new book I purchased"
    b. Save the plan

Task 4: Locate a therapist to schedule an appointment. Let me know when you're done.

1. Find the resources feature
2. Find a therapist in your area from the Therapy for Black Girls Therapist Directory
    a. Search by zip code "28202"
    b. Find "Montina Myers-Galloway" and report whether she is accepting new clients
