# Supplementary material for: Development of a Mobile App to Support Self-management of Anxiety and Depression in African American Women: Usability Study
Source: JMIR Form Res. 2021 Aug 17;5(8):e24393. doi: 10.2196/24393 (PMC8408754; doi:10.2196/24393)
Supplement: Multimedia Appendix 2 [file formative_v5i8e24393_app2.pdf]

COGNITIVE WALKTHROUGH BENCHMARKS

Scenario 1 Tasks

Benchmarks for Tasks		
**Task 1: Find out your levels of anxiety for the past 6 weeks**		
**Steps**	**Correct Action**	**Benchmark**
Step 1: Open the app.	Tap the **TherapyLink** icon	X
Step 2: Find the graphs feature.	Tap the **Graphs** icon	X
Step 3: Find the anxiety checkup graph.	Tap the **Anxiety Checkup History** link	X
Step 4: Report your most recent level of anxiety recorded in the graph (severity and date).	Find and report anxiety severity for February 12	X
	Taps to Complete Task:	**3**
	Time to Complete Task (MM:SS):	**0:13**
**Task 2: Find information on how to manage anxiety**		
**Steps**	**Correct Action**	**Benchmark**
Step 1: Find the information feature.	Use the menu to navigate back to the information feature by taping the hamburger icon or slide right to show menu	X
	Tap the **Back** button in the menu	X
	Tap the **Information** link in the menu	X
Step 2: Find the anxiety information.	Tap the **Anxiety Info** button	X
Step 3: Find the information on how to manage anxiety.	Tap the **Managing Anxiety** tab	X
Step 4: Report the first tip on managing anxiety.	Tap the **Tips** link and report the first tip	X
	Taps to Complete Task:	**6**
	Time to Complete Task (MM:SS):	**0:25**

Benchmarks for Tasks		
**Task 3: Add a new entry to your journal**		
**Steps**	**Correct Action**	**Benchmark**
Step 1: Find the journal feature.	Use the menu to navigate back to the information feature by taping the hamburger icon or slide right to show menu	X
	Tap the **Back** button in the menu	X
	Tap the **Journal** link in the menu	X
Step 2: Create a new journal entry.	Tap the **(+)** button to add a new entry	X
	Tap in the **Title** field and enter "Today was a lot"	X
	Tap in the **Thoughts** field and manually or use the voice-to-text feature to enter, "Today my AP told me that I will be teaching one more section of Algebra in the Spring. I am already overwhelmed with the sections that I have now. I don't know how to tell him that it is going to be too much to manage."	X
	Tap the **Next** button	X
Step 3: Report your feelings.	Tap **Pick Items** to open the dropdown list of feelings	X
	Tap "anxious" and "overwhelmed" as your options to report your feelings	X
	Tap the **Save** button	X
Step 4: Indicate that there is something you can do to make the situation better, or avoid it from happening in the future.	Tap the **Yes** button to answer the question, "Is there anything you can do to make the situation better, or avoid it from happening in the future?	X
Step 5: Report on what you think can be done to make the situation better.	Tap in the field to write out your thoughts and manually or use the voice-to-text feature to enter, "I can email the AP to schedule a meeting to discuss my concerns, and propose that other tasks be taken off my plate if I am going to teach one more class."	X
	Tap the **Next** button	X
Step 6: Save the journal entry.	Review the **Journal Entry Summary** and tap the **Save** button	X
	Taps to Complete Task:	**15**
	Time to Complete Task (MM:SS):	**3:10**

Benchmarks for Tasks		
**Task 4: Locate a therapist to schedule an appointment**		
**Steps**	**Correct Action**	**Benchmark**
Step 1: Find the resources feature	Use the menu to navigate back to the information feature by taping the hamburger icon or slide right to show menu	X
	Tap the **Back** button in the menu	X
	Tap the **Resources** link in the menu	X
Step 2: Find a therapist in your area from the Therapy for Black Girls Therapist Directory	Tap the **Find A Therapist** button	X
	Tap the **Therapy for Black Girls Therapist Directory** button	X
	Tap in the **Your Location** field and enter zip code "33015"	X
	Tap the **Search** button	X
	Scroll down the screen to find "Nakita Charles" and tap the profile	X
	Scroll down the screen and report whether she is accepting new clients	X
	Taps to Complete Task:	**8**
	Time to Complete Task (MM:SS):	**0:56**

Scenario 2 Tasks

Benchmarks for Tasks		
**Task 1: Find out your levels of depression for the past 6 weeks**		
**Steps**	**Correct Action**	**Benchmark**
Step 1: Open the app.	Tap the **TherapyLink** icon	X
Step 2: Find the graphs feature.	Tap the **Graphs** icon	X
Step 3: Find the depression checkup graph.	Tap the **Depression Checkup History** link	X
Step 4: Report your most recent level of depression recorded in the graph (severity and date).	Find and report depression severity for February 12	X
	Taps to Complete Task:	**3**
	Time to Complete Task (MM:SS):	**0:14**
**Task 2: Find information on how to overcome depression**		
**Steps**	**Correct Action**	**Benchmark**
Step 1: Find the information feature.	Use the menu to navigate back to the information feature by taping the hamburger icon or slide right to show menu	X
	Tap the **Back** button in the menu	X
	Tap the **Information** link in the menu	X
Step 2: Find the depression information.	Tap the **Depression Info** button	X
Step 3: Find the information on how to overcome depression.	Tap the **Overcome Depression** tab	X
Step 4: Report the first tip on overcoming depression.	Tap the **Tips** link and report the first tip	X
	Taps to Complete Task:	**6**
	Time to Complete Task (MM:SS):	**0:20**

Benchmarks for Tasks		
**Task 3: Create a self-care plan**		
**Steps**	**Correct Action**	**Benchmark**
Step 1: Find the self-care plan feature.	Use the menu to navigate back to the information feature by taping the hamburger icon or slide right to show menu	X
	Tap the **Back** button in the menu	X
	Tap the **Home** link in the menu	X
	Tap the **Self-Care** icon	X
Step 2: Create a new self-care plan	Tap the **Create Plan** button	X
	Tap the **Start Date** button	X
	Select "March 23, 2020" from the calendar	X
	Tap **OK**	X
	Tap the **End Date** button	X
	Select "March 29, 2020" from the calendar	X
	Tap **OK**	X
	Tap in the first text entry field and enter "Go to yoga class"	X
	Tap in the second text entry field and enter "Have lunch with Ashley"	X
	Tap in the third text entry field and enter "Read one chapter of the new book I purchased"	X
Step 3: Save the self-care plan.	Tap the **Save** button	X
	Taps to Complete Task:	**15**
	Time to Complete Task (MM:SS):	**1:17**
**Task 4: Locate a therapist to schedule an appointment**		
**Steps**	**Correct Action**	**Benchmark**
Step 1: Find the resources feature	Use the menu to navigate back to the information feature by taping the hamburger icon or slide right to show menu	X
	Tap the **Back** button in the menu	X
	Tap the **Resources** link in the menu	X
Step 2: Find a therapist in your area from the Therapy for Black Girls Therapist Directory	Tap the **Find A Therapist** button	X
	Tap the **Therapy for Black Grils Therapist Directory** button	X
	Tap in the **Your Location** field and enter zipcode “28202”	X
	Tap the **Search** button	X
	Scroll down the screen to find “Montina Myers-Galloway” and tap the profile	X
	Scroll down the screen and report whether she is accepting new clients	X
	Taps to Complete Task:	**8**
	Time to Complete Task (MM:SS):	**0:49**
