# Supplementary material for: Development of a Mobile App to Support Self-management of Anxiety and Depression in African American Women: Usability Study
Source: JMIR Form Res. 2021 Aug 17;5(8):e24393. doi: 10.2196/24393 (PMC8408754; doi:10.2196/24393)
Supplement: Multimedia Appendix 3 [file formative_v5i8e24393_app3.pdf]

USABILITY TESTING PARTICIPANT PERFORMANCE VS. BENCHMARK FOR SCENARIO 1 TASKS

Participant Performance vs Benchmark										
**Task 1: Find out your levels of anxiety for the past 6 weeks**										
**Steps**	**Correct Action**	**P1**	**P2**	**P3**	**P4**	**P5**	**P6**	**P7**	**P8**	**Benchmark**
Step 1: Open the app.	Tap the **TherapyLink** icon	X	X	X	X	X	X	X	X	X
Step 2: Find the graphs feature.	Tap the **Graphs** icon	X	X	X	X	X	X	X	X	X
Step 3: Find the anxiety checkup graph.	Tap the **Anxiety Checkup History** link	X	X	X	X	X	X	X	X	X
Step 4: Report your most recent level of anxiety recorded in the graph (severity and date).	Find and report anxiety severity for February 12	X	X	X	X	X	X	X	X	X
	Taps to Complete Task:	**3**	**3**	**3**	**3**	**3**	**3**	**3**	**6**	**3**
	Time to Complete Task (MM:SS):	**0:20**	**0:56**	**1:41**	**0:58**	**1:13**	**0:26**	**0:36**	**1:40**	**0:13**
**Task 2: Find information on how to manage anxiety**										
**Steps**	**Correct Action**	**P1**	**P2**	**P3**	**P4**	**P5**	**P6**	**P7**	**P8**	**Benchmark**
Step 1: Find the information feature.	Use the menu to navigate back to the information feature by taping the hamburger icon or slide right to show menu	X	X		X	X	X		X	X
	Tap the **Back** button in the menu	X	X		X	X	X		X	X
	Tap the **Information** link in the menu	X	X			X	X		X	X
Step 2: Find the anxiety information.	Tap the **Anxiety Info** button	X	X	X	X	X	X	X	X	X
Step 3: Find the information on how to manage anxiety.	Tap the **Managing Anxiety** tab	X	X	X	X	X	X	X	X	X
Step 4: Report the first tip on managing anxiety.	Tap the **Tips** link and report the first tip	X	X	X	X	X	X	X	X	X
	Taps to Complete Task:	**6**	**14**	**6**	**18**	**6**	**37**	**6**	**12**	**6**
	Time to Complete Task (MM:SS):	**0:48**	**1:43**	**1:09**	**1:50**	**1:15**	**4:24**	**0:53**	**2:03**	**0:25**

Participant Performance vs Benchmark										
**Task 3: Add a new entry to your journal**										
**Steps**	**Correct Action**	**P1**	**P2**	**P3**	**P4**	**P5**	**P6**	**P7**	**P8**	**Benchmark**
Step 1: Find the journal feature.	Use the menu to navigate back to the information feature by taping the hamburger icon or slide right to show menu	X	X		X	X			X	X
	Tap the **Back** button in the menu	X	X		X	X			X	X
	Tap the **Journal** link in the menu	X	X			X			X	X
Step 2: Create a new journal entry.	Tap the **(+)** button to add a new entry	X	X	X	X	X	X	X	X	X
	Tap in the **Title** field and enter “Today was a lot”	X	X	X	X	X	X	X	X	X
	Tap in the **Thoughts** field and manually or use the voice-to-text feature to enter, “Today my AP told me that I will be teaching one more section of Algebra in the Spring. I am already overwhelmed with the sections that I have now. I don’t know how to tell him that it is going to be too much to manage.”	X	X	X	X	X	X	X	X	X
	Tap the **Next** button	X	X	X	X	X	X	X	X	X
Step 3: Report your feelings.	Tap **Pick Items** to open the dropdown list of feelings	X	X	X	X	X	X		X	X
	Tap “anxious” and “overwhelmed” as your options to report your feelings	X	X	X	X	X	X		X	X
	Tap the **Save** button	X	X	X	X	X	X	X	X	X
Step 4: Indicate that there is something you can do to make the situation better, or avoid it from happening in the future.	Tap the **Yes** button to answer the question, "Is there anything you can do to make the situation better, or avoid it from happening in the future?	X	X	X	X	X	X			X
Step 5: Report on what you think can be done to make the situation better.	Tap in the field to write out your thoughts and manually or use the voice-to-text feature to enter, “I can email the AP to schedule a meeting to discuss my concerns, and propose that other tasks be taken off my plate if I am going to teach one more class.”	X	X	X	X	X				X
	Tap the **Next** button	X	X	X	X	X	X			X
Step 6: Save the journal entry.	Review the **Journal Entry Summary** and tap the **Save** button	X	X	X	X	X			X	X
	Taps to Complete Task:	**17**	**18**	**18**	**17**	**19**	**15***	**9***	**13***	**15**
	Time to Complete Task (MM:SS):	**4:07**	**2:48**	**6:10**	**3:23**	**4:50**	**4:23***	**4:17***	**3:10***	**3:10**

*Participant was unable to fully complete task.

Participant Performance vs Benchmark

Task 4: Locate a therapist to schedule an appointment

Steps	Correct Action	P1	P2	P3	P4	P5	P6	P7	P8	Benchmark
Step 1: Find the resources feature	Use the menu to navigate back to the information feature by taping the hamburger icon or slide right to show menu	X	X	X	X	X			X	X
	Tap the **Back** button in the menu	X	X	X	X	X			X	X
	Tap the **Resources** link in the menu	X	X			X				X
Step 2: Find a therapist in your area from the Therapy for Black Girls Therapist Directory	Tap the **Find A Therapist** button	X	X	X	X	X	X	X	X	X
	Tap the **Therapy for Black Girls Therapist Directory** button	X	X	X	X	X	X	X	X	X
	Tap in the **Your Location** field and enter zip code "33015"	X	X	X		X	X	X	X	X
	Tap the **Search** button	X	X	X	X	X	X	X	X	X
	Scroll down the screen to find "Nakita Charles" and tap the profile	X	X	X	X	X	X	X	X	X
	Scroll down the screen and report whether she is accepting new clients	X	X	X	X	X	X	X	X	X
	Taps to Complete Task:	**10**	**15**	**11**	**35**	**11**	**13**	**10**	**14**	**8**
	Time to Complete Task (MM:SS):	**2:04**	**1:42**	**2:10**	**3:21**	**1:43**	**2:13**	**1:46**	**3:14**	**0:56**
