# Supplementary material for: Development of a Mobile App to Support Self-management of Anxiety and Depression in African American Women: Usability Study
Source: JMIR Form Res. 2021 Aug 17;5(8):e24393. doi: 10.2196/24393 (PMC8408754; doi:10.2196/24393)
Supplement: Multimedia Appendix 4 [file formative_v5i8e24393_app4.pdf]

USABILITY TESTING PARTICIPANT PERFORMANCE VS. BENCHMARK FOR SCENARIO 2 TASKS

Participant Performance vs Benchmark									
**Task 1: Find out your levels of depression for the past 6 weeks**									
**Steps**	**Correct Action**	**P9**	**P10**	**P11**	**P12**	**P13**	**P14**	**P15**	**Benchmark**
Step 1: Open the app.	Tap the **TherapyLink** icon	X	X	X	X	X	X	X	X
Step 2: Find the graphs feature.	Tap the **Graphs** icon	X	X	X	X	X	X	X	X
Step 3: Find the depression checkup graph.	Tap the **Depression Checkup History** link	X	X	X	X	X	X	X	X
Step 4: Report your most recent level of depression recorded in the graph (severity and date).	Find and report depression severity for February 12	X	X	X	X	X	X	X	X
	Taps to Complete Task:	**3**	**3**	**5**	**3**	**3**	**3**	**6**	**3**
	Time to Complete Task (MM:SS):	**0:48**	**0:27**	**0:37**	**0:31**	**0:30**	**0:37**	**0:44**	**0:14**
**Task 2: Find information on how to overcome depression**									
**Steps**	**Correct Action**	**P9**	**P10**	**P11**	**P12**	**P13**	**P14**	**P15**	**Benchmark**
Step 1: Find the information feature.	Use the menu to navigate back to the information feature by taping the hamburger icon or slide right to show menu					X	X	X	X
	Tap the **Back** button in the menu					X	X	X	X
	Tap the **Information** link in the menu					X	X	X	X
Step 2: Find the depression information.	Tap the **Depression Info** button	X	X	X	X	X	X	X	X
Step 3: Find the information on how to overcome depression.	Tap the **Overcome Depression** tab	X	X	X	X	X	X	X	X
Step 4: Report the first tip on overcoming depression.	Tap the **Tips** link and report the first tip	X	X	X	X	X	X	X	X
	Taps to Complete Task:	**14**	**12**	**6**	**9**	**6**	**6**	**11**	**6**
	Time to Complete Task (MM:SS):	**1:38**	**2:14**	**0:28**	**1:50**	**0:45**	**1:24**	**1:11**	**0:20**

Participant Performance vs Benchmark									
Task 3: Create a self-care plan									
**Steps**	**Correct Action**	**P9**	**P10**	**P11**	**P12**	**P13**	**P14**	**P15**	**Benchmark**
Step 1: Find the self-care plan feature.	Use the menu to navigate back to the information feature by taping the hamburger icon or slide right to show menu					X	X		X
	Tap the **Back** button in the menu					X	X		X
	Tap the **Home** link in the menu					X	X		X
	Tap the **Self-Care** icon	X	X	X	X	X	X	X	X
Step 2: Create a new self-care plan	Tap the **Create Plan** button	X	X	X	X	X	X	X	X
	Tap the **Start Date** button	X	X	X	X	X	X	X	X
	Select "March 23, 2020" from the calendar	X	X	X	X	X	X	X	X
	Tap **OK**	X	X	X	X	X	X	X	X
	Tap the **End Date** button	X	X	X	X	X	X	X	X
	Select "March 29, 2020" from the calendar	X	X	X	X	X	X	X	X
	Tap **OK**	X	X	X	X	X	X	X	X
	Tap in the first text entry field and enter "Go to yoga class"	X	X	X	X	X	X	X	X
	Tap in the second text entry field and enter "Have lunch with Ashley"	X	X	X	X	X	X	X	X
	Tap in the third text entry field and enter "Read one chapter of the new book I purchased"	X	X	X	X	X	X	X	X
Step 3: Save the self-care plan.	Tap the **Save** button	X	X	X	X	X	X	X	X
	Taps to Complete Task:	**21**	**23**	**19**	**21**	**21**	**17**	**16**	**15**
	Time to Complete Task (MM:SS):	**2:08**	**1:57**	**1:27**	**1:42**	**2:47**	**2:08**	**1:17**	**1:17**

Participant Performance vs Benchmark									
Task 4: Locate a therapist to schedule an appointment									
**Steps**	**Correct Action**	**P9**	**P10**	**P11**	**P12**	**P13**	**P14**	**P15**	**Benchmark**
Step 1: Find the resources feature	Use the menu to navigate back to the information feature by taping the hamburger icon or slide right to show menu				X	X	X		X
	Tap the **Back** button in the menu				X	X	X		X
	Tap the **Resources** link in the menu					X	X		X
Step 2: Find a therapist in your area from the Therapy for Black Girls Therapist Directory	Tap the **Find A Therapist** button	X	X	X	X	X	X	X	X
	Tap the **Therapy for Black Grils Therapist Directory** button	X	X	X	X	X	X	X	X
	Tap in the **Your Location** field and enter zipcode "28202"	X	X	X	X	X	X	X	X
	Tap the **Search** button	X	X	X	X	X	X	X	X
	Scroll down the screen to find "Montina Myers-Galloway" and tap the profile	X	X	X	X	X	X	X	X
	Scroll down the screen and report whether she is accepting new clients	X	X	X	X	X	X	X	X
	Taps to Complete Task:	**9**	**13**	**7**	**9**	**9**	**9**	**11**	**8**
	Time to Complete Task (MM:SS):	**1:24**	**2:09**	**1:07**	**1:26**	**1:22**	**1:55**	**1:30**	**0:49**
